# Is an extensive allergy diagnosis workup needed in all cases of suspected hypersensitivity to beta-lactams in children?

**DOI:** 10.1186/2045-7022-4-S3-P141

**Published:** 2014-07-18

**Authors:** Eunice Dias de Castro, Fabrícia Carolino, Josefina Rodrigues Cernadas

**Affiliations:** 1 CH São João, Immunoallergology Department, Portugal

## Introduction

Beta-lactams (BL) are one of the most prescribed antibiotics (AB) class. They are frequently implicated in hypersensitivity reactions (HSR), with common skin involvement. However, infectious conditions might also induce cutaneous lesions, especially in children, and they constitute an important differential diagnosis to BL HSR.

## Aims

To characterize suspected BL HSR and analyse the prevalence of allergic reactions in our study population.

## Methods

We retrospectively reviewed the clinical data from all children (<18 years-old) referred to our Allergology Department for suspected BL HSR between 2011 and 2013.

## Results

Eighty children (56.2% females) with a mean age of 6.7 yrs (SD=4.035) where evaluated for 83 suspected BL HSR. The mean time elapsed between the reaction and the clinical assessment was of 3.1 yrs (SD=3.36). The most frequently implicated drugs were amoxicillin/clavulanate (54.2%) and amoxicillin (32.5%). BL were prescribed for tonsillitis in 32.5%, otitis in 21.7% and upper airways infection in 19.3%. Cutaneous lesions were the most frequently reported (94%) manifestations, with maculopapular exanthema (MPE) being the most common. In 45 reactions (54.2%), the time of onset was unknown; in 4.8% the reaction was immediate and in the remaining cases non immediate (41%). In 23 cases (27.7%) there was no history of previous exposure to the suspected drug; 15 children had previous exposure with a similar reaction. In the group of patients with immediate reactions or unknown time of onset (n=49), determination of specific IgE (sIgE) for penicillin G, V and amoxicillin was performed in 43 (87.8%), and skin prick tests in 42 patients (85.7%), all with negative results. Of all patients, 16 (20%) underwent intradermal testing (IDT) and only one patient had a positive result with PPL 1/1; late reading was done in 3 cases, all negative. Oral provocation test (OPT) with the suspected BL (single or continued at home) was performed in 74 cases (89.2%) with positive result in 6 patients (8.1%), all of them exclusively with late cutaneous involvement.

## Discussion

Suspected BL HSR was only confirmed in 8.1%. Although MPE caused by BL treatment in children is rare, there are no clinical distinctive characteristics from MPE related to infections, making allergy diagnosis workup mandatory. The authors raise the question of the need of sIgE determination and skin testing in less severe reactions, instead of performing directly the OPT.

